# A pharmacist-led system-change smoking cessation intervention for smokers admitted to Australian public hospitals (GIVE UP FOR GOOD): study protocol for a randomised controlled trial

**DOI:** 10.1186/1745-6215-14-148

**Published:** 2013-05-21

**Authors:** Dennis Thomas, Michael J Abramson, Billie Bonevski, Simone Taylor, Susan Poole, Gregory R Weeks, Michael J Dooley, Johnson George

**Affiliations:** 1 Centre for Medicine Use and Safety, Faculty of Pharmacy and Pharmaceutical Sciences, Monash University (Parkville Campus), 381 Royal Parade, Parkville, VIC, 3052, Australia; 2 Department of Epidemiology & Preventive Medicine, Faculty of Medicine, Nursing and Health Sciences, Monash University, The Alfred, Melbourne, VIC, 3004, Australia; 3 School of Medicine & Public Health, University of Newcastle, Callaghan, Level 5 McAuley Centre, Calvary Mater, NSW, 2308, Australia; 4 Pharmacy Department, Austin Health, 145 Studley Road, Heidelberg, VIC, 3084, Australia; 5 Pharmacy Department, The Alfred, Commercial Road, Prahran, VIC, 3181, Australia; 6 Pharmacy Department, Barwon Health, Bellarine Street, Geelong, VIC, 3220, Australia

**Keywords:** Smoking, Tobacco dependence, Quitting, Hospital, Randomised controlled trial, Pharmacist, System change

## Abstract

**Background:**

Intensive smoking cessation interventions initiated during hospitalisation are effective, but currently not widely available. Strategies are needed to integrate smoking cessation treatment into routine inpatient care. Pharmacist-led interventions for smoking cessation are feasible and efficacious in both ambulatory and community pharmacy settings. However, there is a lack of evidence from large scale studies of the effectiveness of pharmacist guided programs initiated during patient hospitalisation in achieving long-term abstinence. This study aims to evaluate the effectiveness of a pharmacist-led system change intervention initiated during hospitalisation in Australian public hospitals.

**Methods/design:**

A multi-centre, randomised controlled trial will be conducted with 12 months follow-up. Smokers, 18 years or older, will be recruited from the wards of three Victorian public hospitals. Participants will be randomly assigned to a usual care or intervention group using a computer generated randomisation list. The intervention group will receive at least three smoking cessation support sessions by a trained pharmacist: the first during the hospital stay, the second on or immediately after discharge and the third within one month post-discharge. All smoking cessation medications will be provided free of charge during the hospital stay and for at least one week after discharge. Participants randomised to usual care will receive the current care routinely provided by the hospital. All measurements at baseline, discharge, one, six and 12 months will be performed by a blinded Research Assistant. The primary outcome measures are carbon monoxide validated 7-day point prevalence abstinence at six and 12 months.

**Discussion:**

This is the first large scale study to develop and test a pharmacist-led system change intervention program initiated during patient hospitalisation. If successful, the program could be considered for wider implementation across other hospitals.

**Trial registration:**

ACTRN12612000368831

## Background

Tobacco smoking continues to be the leading cause of preventable morbidity and mortality worldwide [1]. According to the World Health Organisation, tobacco use claims almost six million lives every year and, if the current trend continues, by 2030 tobacco will cause more than eight million deaths per year worldwide [1]. Smoking contributes to more than 15,500 deaths (11.7% of total deaths) and 7.8% of the total burden of disease and injury in Australia [2]. The annual smoking-related costs to the society are estimated at $31.5 billion [3]. Despite this, one in seven Australians 14 years old and over continues to smoke every day [4].

Hospitalisation may provide an ideal opportunity for health professionals to assist people to quit smoking and it is a potential teachable moment for smoking cessation [5,6]. At a time of perceived vulnerability to negative health outcomes, individuals may want to be more aware about the health risks. They may also be more receptive to smoking cessation messages and interventions [7,8]. Many Australian hospitals have implemented policies whereby smoking is not permitted indoors or within their boundaries outdoors [9]. Health benefits to the community are likely to be more pronounced if smoking bans are accompanied by supportive services to assist smokers to quit [10].

Clinical practice guidelines for treating tobacco use and dependence recommend that healthcare institutions develop plans to support the consistent and effective identification, documentation and treatment of tobacco users [11]. Ginn *et al*. has described the initiatives of an interdisciplinary group at an urban academic medical centre in the United States of America (USA) in the development and implementation of a tobacco cessation protocol [12]. The protocol focused on admission assessment, education, and provision of standing orders for medication treatment for nicotine withdrawal and/or tobacco cessation therapy during the inpatient encounter and referral for outpatient counselling on discharge.

High intensity behavioural interventions that are initiated during the hospital stay and include at least one month of follow-up after discharge have been found to increase smoking cessation among hospitalised patients by 37% [13]. Despite the availability of evidence to support high intensity behavioural interventions and best practice guidelines on cessation support for hospital inpatients, low levels of smoking cessation care are provided [9,14,15]. For example, a survey of patients with a smoking history admitted to a Victorian tertiary hospital found that almost half were interested in starting a smoking cessation program whilst in hospital. Despite this, only one in five had had any discussion with a health professional regarding options to assist with quitting in hospital [16]. The barriers to providing effective smoking cessation include a lack of support from the organisation, perceived patient objections, a lack of systems to identify smokers, a lack of time and skill, perceived inability to change care practices, a perceived lack of efficacy of smoking cessation treatments and the cost of providing care [17].

Other initiatives are required that include a system change approach to address the multidimensional problems associated with smoking care provision [18]. Having a dedicated and trained professional for screening, documenting and providing smoking cessation support may be an effective approach. Such initiatives are currently not available in Australia.

Pharmacist-led interventions for smoking cessation have been shown to be feasible and efficacious in both hospital outpatient and community pharmacy settings [19-22]. However, there is no evidence from large scale studies for the effectiveness of pharmacy interventions initiated in hospitalised patients that achieve long-term abstinence. Work has commenced in the USA with the Consortium of Hospitals Advancing Research on Tobacco (CHART) network assessing the effectiveness and cost-effectiveness of a number of projects initiated during hospitalisation and continued post-discharge. This project is expected to include 10,000 hospitalised smokers from 20 hospitals in the USA [10]. However, this project does not include an intervention with significant pharmacy involvement.

We hypothesise that a multi-disciplinary, system change, high intensity behavioural intervention led by hospital pharmacists offering pharmacotherapy and non-pharmacotherapy as needed, that begins during a hospital stay with at least one month of supportive contact after discharge has the potential to achieve long-term abstinence.

### Objectives

The primary aim of the study is to determine the effectiveness of a pharmacist-led system change intervention (‘GIVE UP FOR GOOD’) compared to usual care on biochemically verified 7-day point prevalence abstinence at six months and 12 months.

The secondary objectives are

1. To evaluate the effectiveness of the ‘GIVE UP FOR GOOD’ intervention compared to usual care on self-reported continuous abstinence at discharge and at one, six and 12 months post-discharge.

2. To evaluate the effectiveness of the ‘GIVE UP FOR GOOD’ intervention compared to usual care on self-reported 24 hour, 7-day and 30-day point prevalence abstinence at one, six and 12 months post-discharge.

## Methods/design

**Figure 1:**
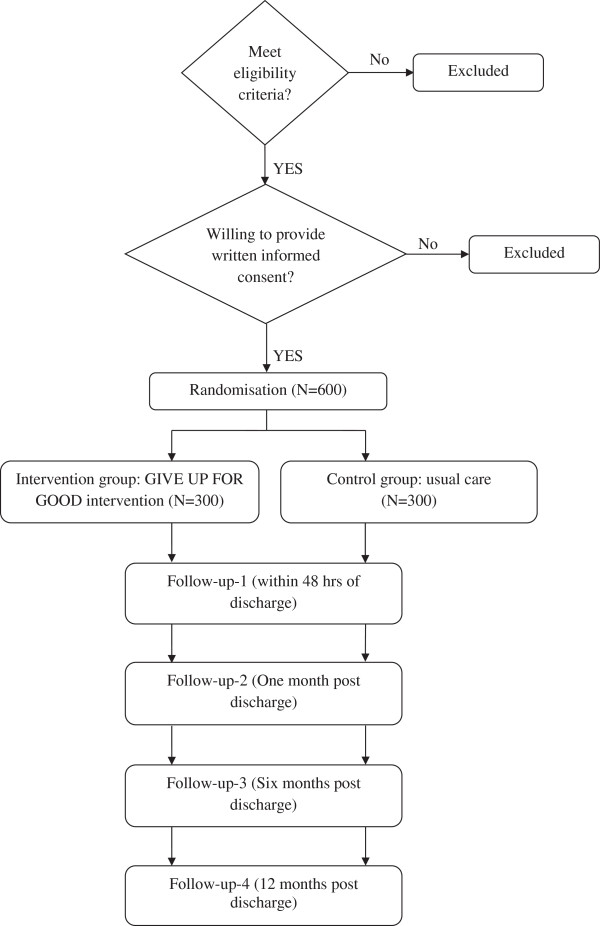
Flow diagram of GIVE UP FOR GOOD study.

This is a randomised, multi-centre, single blinded study. Participants will be recruited from the inpatient wards of three Victorian public hospitals: The Alfred, Austin Health and Barwon Health. Each participant will be screened for eligibility at baseline. Eligible participants will be randomised to either the intervention or usual care group, and complete four additional follow-up interviews over a period of 12 months. The ‘GIVE UP FOR GOOD’ intervention will be delivered over the course of at least three sessions (Figure 1: Study Flow Diagram).

### Inclusion and exclusion criteria

Patients eligible for the trial are 18 years old or older, are smokers at the time of hospital admission, and are available for follow-up on discharge, and up to 12 months post-discharge. Exclusion criteria include physical or mental inability to participate in the study, inability to provide written informed consent, inability to communicate in English, terminal illness, pregnancy or on another active smoking cessation therapy or program at the time of hospital admission (pharmacotherapy including nicotine replacement therapy (NRT) or active involvement in a smoking cessation program in the last seven days prior to the hospital admission with support from a trained counsellor, health professional or service provider).

### Study conduct

Eligible participants will be identified through active screening of admission, nursing and medical notes by a Research Assistant (RA) employed at each site. Ward staff, including doctors, nurses, pharmacists and physiotherapists will be informed of the research project and asked to refer all patients identified as current smokers to the RA. The RA will describe the project to each potential participant, provide a plain language statement and answer any questions. If he/she is interested in participating, written informed consent will be sought before proceeding with the baseline interview. All participants will be referred to a study pharmacist for randomisation after baseline data collection.

### Randomisation: allocation, concealment and sequence generation

At each site, patients meeting all the entry criteria will be randomised by the study pharmacist to either of the study arms, using a computer generated randomisation list. Stratified, block randomisation with random block sizes of four and eight will be used. The study pharmacist will be kept unaware of block length to avoid the predictability of treatment allocation. Participants will be stratified into two groups using the Heaviness of Smoking Index (HSI) [23]: heavy smokers (HSI score ≥4) and light smokers (HSI Score ≤3). Equal proportions of heavy and light smokers will be approached.

Sealed opaque envelopes will be used for the concealment of allocation. The study pharmacist will assess the participants’ nicotine dependence using HSI and categorise them into the respective stratum. The study pharmacist will then sequentially select and open an envelope corresponding to the stratum to identify treatment allocation and the randomisation number, unique for each participant.

### Usual care group

Participants randomised to the usual care (control) group will continue to receive routine care provided by the hospital. They may receive brief counselling by hospital staff and/or free NRT or pharmacotherapy during their hospital stay as per hospital policy. All three participating hospitals have a ‘smoke free’ policy; however, there may be differences between sites in the extent to which staff offer support to smokers to quit as part of routine care. The support provided to the usual care group at each site will be recorded and reported.

### Intervention group

Participants randomised to the intervention group will receive the ‘GIVE UP FOR GOOD’ smoking cessation intervention coordinated by the study pharmacist. The conceptual framework for the intervention is based on the systems change approach of Fiore *et al*. [24], which has six systems-level strategies to facilitate treatment of tobacco dependence:

1. Implement a system of identifying smokers;

2. Provide education and resources to promote provider intervention;

3. Dedicate staff to provide smoking cessation services;

4. Promote hospital policies that support and provide tobacco dependence services;

5. Include tobacco dependence treatments (both counselling and pharmacotherapies) identified as effective; and

6. Reimburse health care providers for delivery of effective tobacco dependence treatments and include these services among their defined duties.

In GIVE UP FOR GOOD, trained and dedicated hospital pharmacists take the lead role in providing smoking cessation support to inpatients at each participating hospital. The pharmacist will record each participant’s smoking status in the medical records and provide cessation support including counselling and pharmacotherapy.

### Intervention procedures

All the intervention pharmacists will complete a two day smoking cessation training program for health professionals conducted by the Lung Health Promotion Centre (LHPC) at The Alfred, Melbourne, Australia. Participants randomised to the ‘GIVE UP FOR GOOD’ program will receive a series of smoking cessation counselling sessions by one of the two specially trained pharmacists at each hospital over the course of at least three sessions: the first during the hospital stay, the second on discharge or immediately after discharge and the third within one month post-discharge. The consistency of intervention across the sites will be maintained by using standardised treatment algorithms and procedures, and regular discussions. Pharmacists will also be regularly attending smoking cessation update sessions conducted by the LHPC at approximately six month intervals.

### Intervention 1 (after baseline data collection)

Study pharmacists will review the participants’ medical and medication history in conjunction with their smoking history, nicotine addiction, stages of change, co-morbidities, quit attempts and outcomes in the past. The study pharmacist will then discuss with each participant the available options for quitting, including cognitive and behavioural strategies and/or pharmacotherapy. Following motivational interviewing, a QUIT Plan [25] will be prepared for each participant. Resources such as QUIT Pack®, QUIT Line® and QUIT Coach® and referral to other staff such as doctors, nurses and dieticians will be used, where appropriate. If prescription medications are required to assist smoking cessation, this will be discussed with the treating medical staff and provided free of charge during the hospital stay. Over-the-counter NRT products may be initiated by the study pharmacists as required and at the patient’s discretion. Intervention 1 will take approximately 30 minutes.

### Intervention 2 (immediately before or after hospital discharge)

The study pharmacist will reinforce the importance of quitting, and discuss relapse and relapse prevention strategies with the participant. A smoking treatment summary and the discharge plan will be sent to the participant’s general practitioner and community pharmacist with instructions for post-discharge management (including non-pharmacological treatment). An appointment with the primary healthcare professionals will be made on the participant’s behalf. If participants required pharmacotherapy for smoking cessation during their hospital stay, they will receive at least one week’s supply free of charge at the time of discharge. Intervention 2 will take approximately 15 minutes.

### Intervention 3 (within one month after hospital discharge)

The study pharmacist will follow-up the participant approximately four weeks post-discharge (by telephone or mail) to emphasise the importance of long-term abstinence and remind those who have not been reviewed by their primary health professionals after discharge to seek ongoing smoking cessation support. Intervention 3 will take approximately 10 minutes.

### Data collection and follow-up

Baseline data collection will be at the time of recruitment. All participants will be followed up for a period of 12 months from hospital discharge. Telephone, mail or face-face follow-up interviews will be conducted at one, six and 12 months post-discharge.

General demographics including age, gender, nationality, language, education, employment, marital status, income, living arrangement and possession of any concession card will be collected at baseline. Medical and medication history will be obtained from the patients’ notes. Smoking-related information including current smoking status (daily or occasional smoker), age at which smoking started and smoking habits of friends and housemates also will be captured. In addition, smoking-related data, such as smoking habits, money spent on cigarettes, previous smoking cessation attempts and outcomes, methods used for cessation and difficulties faced during past quit attempts, will be collected. Preferred methods of cessation, medications, strategies and facilitators to assist quitting, discussions about smoking cessation with health professionals during the present hospitalisation and in the past, motivation and confidence to give up smoking will also be determined. Participant’s satisfaction with the current services received will be evaluated using a five point scale (1- very dissatisfied to 5- very satisfied). Charlson’s Co-morbidity Index [26] will be used for assessing the co-morbid conditions.

The study will use the following validated scales:

•Heaviness of smoking index (HSI): [23] This is a two item scale to assess nicotine dependence based on time to first cigarette of the day and number of cigarettes smoked per day.

•Patient Health Questionnaire (PHQ-2): [27] This two item scale will be used to assess the frequency of depressed mood and inability to experience pleasure. Each item will be scored on a four point scale ranging from ‘not at all’ to ‘nearly every day’.

•Smoking self-efficacy scale: [28] Self-efficacy will be assessed using nine items, in order to determine temptation to smoke in various situations. Each item is answered on a five point scale ranging from ‘Not at all tempted’ to ‘Extremely tempted’ to smoke. Higher scores indicate greater smoking temptation.

•Readiness to quit ladder: [29] This scale has 10 response options that assess motivation along a continuum, from ‘not considering quitting in the near future’ to ‘have already quit smoking’. Higher scores suggest greater motivation to quit smoking.

•Short Form (SF-8) quality of life questionnaire: [30] This eight item scale will be used to assess general health-related quality of life. The scale has domains on physical and mental health. The items represent physical functioning, role-physical (role limitations due to physical health problems), bodily pain, general health, vitality, social functioning, role-emotional (role limitations due to personal or emotional problems) and mental health. A higher score indicates better health.

•Visual Analogue Scales (VAS) for motivation and confidence: These scales will be used to assess participants’ motivation and confidence to give up smoking on a 10 point scale, with one being ‘very low’ and 10 being ‘very high’.

The readiness to quit ladder and HSI will be used at each follow-up. The smoking self efficacy scale and PHQ-2 will be used at each follow-up, except follow-up 1. The quality of life (SF-8) will be assessed at baseline and at the end of the study.

### Blinding

All assessments will be conducted by a RA blinded to treatment allocation. All possible measures will be taken to prevent the revealing of treatment allocation to the RA. Any accidental unblinding (for example, participant revealing details of the intervention during a follow-up interview) will be documented and reported.

### Primary endpoint

The primary endpoints are biochemically verified 7-day point prevalence abstinence at six and 12 months. Participants who self-report abstinence of at least seven consecutive days (7-day point prevalence) at six and 12 months will be asked to perform a carbon monoxide (CO) breath test. It will be measured using a hand-held piCO+ Smokerlyzer (Bedfont Scientific, Maidstone, Kent, England) [31]. Participants will be requested to inhale and hold their breath for 15 seconds before exhaling into the analyser. An exhaled CO level of six ppm is recommended by the manufacturer for distinguishing smokers and non-smokers. A non-smoker is expected to have a CO level of 0 to 6 ppm, a mildly dependent smoker 7 to 15 ppm and a strongly addicted smoker over 15 ppm. The instrument will be calibrated regularly according to the manufacturer’s specifications.

Participants who report having smoked more than five cigarettes in the previous 30 days at the six month follow-up or in the previous six months at the 12 month follow-up will be regarded as smokers and will be excluded from the CO breath test. A participant with a CO level ≤6 ppm will be considered abstinent. If there is a conflict between self-reported smoking status and the CO breath test result, the latter will be taken as the ‘gold standard’. Participants who fail to complete a follow-up will be considered to be continuing smokers at that point.

### Secondary endpoints

The secondary outcome measures are 1) Participant self-reported continuous abstinence (defined as abstinence between quit day and a follow-up point) at one, six and 12 months. 2) Self-reported 24-hour, 7-day and 30-day point prevalence abstinence (defined as prevalence of abstinence during a time window immediately preceding the follow-up) at one, six and 12 months. 3) Self-reported cigarette consumption at baseline, one, six and 12 months. 4) Self-reported spending on cigarettes at baseline, one, six and 12 months. 5) Response to the various validated scales including HSI, PHQ-2, smoking self-efficacy scale, readiness to quit ladder, SF-8 and VAS.

### Sample size

The abstinence rate at six months was 28% in studies where trained community pharmacists offered counselling in conjunction with pharmacotherapy, whereas it ranged between 8% and 11.8% in the control group [20,21]. Using a conservative approach based on these findings, with 250 participants per group, this trial will have 95% power to detect a 13% difference in the proportion of quitters (25% versus 12%) with a two sided *P*-value of 0.05. To allow for a potential drop-out of 20%, 600 patients will be recruited in total. Our aim is to recruit the required number of participants from three Victorian hospitals over 12 months, 200 smokers from each hospital (that is, 100 usual care and 100 intervention).

### Statistics and data analysis

Data will be assessed for normality and analysed using appropriate statistical tests. The baseline demographic and clinical characteristics will be summarised using proportions, mean and standard deviation, or median and interquartile range, as appropriate.

Base-line comparisons: characteristics of study participants in the intervention and usual care groups will be compared using the chi-square test for categorical variables and the Student’s *t*-test or a non-parametric equivalent (for example, the Mann–Whitney *U* test) for continuous and discrete variables.

Comparisons between the intervention and usual care group will be performed (both adjusted and unadjusted) for the known confounders. Analysis of primary outcome will involve comparing the changes in quit rates between the two groups. Multivariable analysis will be used to compare outcomes between the two treatment groups while adjusting for prognostic variables and potential confounders. Analysis of secondary outcomes will be conducted using standard statistical procedures applicable to the categorical, continuous or discrete variables. All the statistical tests will be interpreted with a significance level of 5% (two-tailed).

Data will be analysed according to intention-to-treat (ITT) principles. All randomised participants will be included in the analysis and those lost to follow-up will be regarded as smokers. Participants who die during the study will be excluded from the analysis [32]. In addition to ITT analysis, a per protocol analysis also will be performed.

### Ethics

The trial will be conducted in compliance with the protocol, the principles of Good Clinical Practice (GCP) [33], the National Health and Medical Research Council’s (NHMRC) National Statement on Ethical Conduct in Human Research (2007) [34] and the Australian Code for the Responsible Conduct of Research (2007) [35]. This study has been approved by the Human Research Ethics Committees of all three participating hospitals and Monash University. Written informed consent will be obtained from each participant at the time of enrolment.

## Discussion

This is the first multi-centre study to develop and evaluate a pharmacist-led system change intervention program in hospitalised patients. This is also the first large scale study to explore the effectiveness of pharmacist interventions in achieving long-term abstinence among inpatients in Australian hospitals. This project is endorsed by the Chief Executive Officers and the Directors of Pharmacy of the participating hospitals which will ensure support from hospital staff, thus facilitating the recruitment of participants and conduct of the study. The three participating hospitals have prohibited smoking in their premises. Such a smoke free environment will promote cessation in both usual care and intervention groups equally. Smoking bans with supportive services to assist smokers to quit with or without pharmacotherapy are likely to produce more health benefits to the community.

Experience from the implementation and evaluation of the ‘GIVE UP FOR GOOD’ intervention at three sites will guide the provision of smoking cessation support for hospital inpatients. If cost effective, the findings of this study could influence policy changes leading to allocation of more resources to support smoking cessation initiatives through public hospitals in Australia. If successful, the program could be implemented across other hospitals in Australia and overseas with minimal or no changes.

## Trial status

The trial is currently in the recruitment phase.

## Abbreviations

CO: Carbon monoxide; HSI: Heaviness of smoking index; ITT: Intention-To-Treat; LHPC: Lung Health Promotion Centre; NRT: Nicotine replacement therapy; PHQ: Patient Health Questionnaire; RA: Research assistant; VAS: Visual analogue scale

## Competing interests

The study is supported by an investigator initiated research (IIR) grant from Pfizer. However, Pfizer was not involved in the design of the study, protocol development or implementation and will not be involved in the analysis and publication of findings. Recommendations for pharmacotherapy will be evidence-based and according to guidelines based on a participant’s nicotine dependence and participant preference. Professor Abramson was a member of the Scientific Committee for a workshop on an unrelated topic that was sponsored by GlaxoSmithKline, but did not receive any honorarium.

## Authors’ contributions

JG conceived the research idea and developed it with input from the other chief investigators, MA, BB, ST, SP, GW and MD, and secured research funding. DT is a PhD scholar working on the project under the supervision of JG, MA and BB. All the investigators contributed to all phases of the study including study design, protocol development and finalisation of manuscript. All authors have reviewed this manuscript and have approved the final protocol.
